# Amphetamine-Induced Ischemic Colitis: A Case Report

**DOI:** 10.7759/cureus.92226

**Published:** 2025-09-13

**Authors:** Soham Bhanvadia, Shivam Bhanvadia, Hadass Fuerst

**Affiliations:** 1 Family Medicine, Midwestern University Arizona College of Osteopathic Medicine, Glendale, USA; 2 Family Medicine, Abrazo Community Health Network, Phoenix, USA

**Keywords:** abdominal pain, amphetamine use, elderly patient, gastrointestinal ischemia, ischemic colitis

## Abstract

We report a rare case of ischemic colitis secondary to amphetamine use in a 72-year-old woman, highlighting an uncommon etiology in an elderly patient. The patient, with a medical history of hypertension, deep vein thrombosis, scoliosis, and polysubstance abuse, presented to the emergency department with abdominal pain, diarrhea, and melena that had persisted for the past 24 hours. On initial examination, she was found to be tachycardic, tachypneic, and hypertensive. Diagnostic evaluation included laboratory tests, computed tomography (CT) imaging revealing severe colitis affecting the distal transverse, descending, and sigmoid colon, and a urine drug screen that was positive for amphetamines. Gastroenterology was consulted, and the diagnosis of ischemic colitis secondary to amphetamine use was confirmed. The patient was treated with supportive care and demonstrated significant clinical improvement over the next three days, with resolution of symptoms at follow-up one week later. This case emphasizes the need for healthcare providers to consider amphetamine use as a potential cause of ischemic colitis, regardless of age, and underscores the need for heightened clinical vigilance amid rising amphetamine use. Recognition of this association may broaden diagnostic considerations and guide timely, supportive management to improve outcomes in similar patients.

## Introduction

Ischemic colitis is typically seen in elderly patients and results from a reduction of blood flow to the colon, leading to inflammation and tissue damage [1]. It classically presents with sudden left lower abdominal cramping, an urgent need to defecate, and passage of bright red blood per rectum or bloody diarrhea within 24 hours [1]. While several factors can cause or predispose someone to ischemic colitis, including atherosclerosis, hypotension, and hypercoagulable states, its occurrence due to amphetamine use is rare [2]. Amphetamine-induced ischemia is thought to arise from intense vasoconstriction and microvascular compromise, mimicking the pathophysiology of other sympathomimetic agents such as cocaine [2,3]. Reported cases of amphetamine-associated ischemic colitis have primarily involved patients under the age of 50 [2]. Recent trends reveal a troubling rise in amphetamine use, particularly methamphetamine, which is now considered the second most widely used illicit drug globally, with an estimated annual prevalence of 0.4% [3]. As substance use patterns shift across age groups, it is critical that clinicians remain alert to atypical presentations of well-known conditions [4]. We present a case of ischemic colitis secondary to amphetamine use in a 72-year-old woman managed successfully through supportive care. To our knowledge, this represents one of the few documented cases of amphetamine-associated ischemic colitis in a patient over the age of 70, highlighting the evolving demographic of drug-related gastrointestinal pathology and underscoring the importance of recognizing this presentation in older patients [2].

## Case presentation

A 72-year-old woman with a history of hypertension, deep vein thrombosis (DVT), scoliosis, and polysubstance abuse presented to the emergency department (ED) with abdominal pain, diarrhea, and melena for the past 24 hours. She denied chest pain, shortness of breath, nausea, vomiting, leg pain, or other concerning symptoms at the time of presentation. She also denied any history of gastrointestinal (GI) disease or a family history of GI disease. Home medications included cholecalciferol, hydrochlorothiazide, and lisinopril. The patient’s substance use history included fentanyl, methamphetamine, and marijuana use. According to her sister, the patient began using drugs one year ago following a ground-level fall that required opioids for pain management after a total hip arthroplasty. Since then, the patient had attended multiple parties and used various illicit substances. She denied any tobacco or alcohol use.

**Figure 1:**
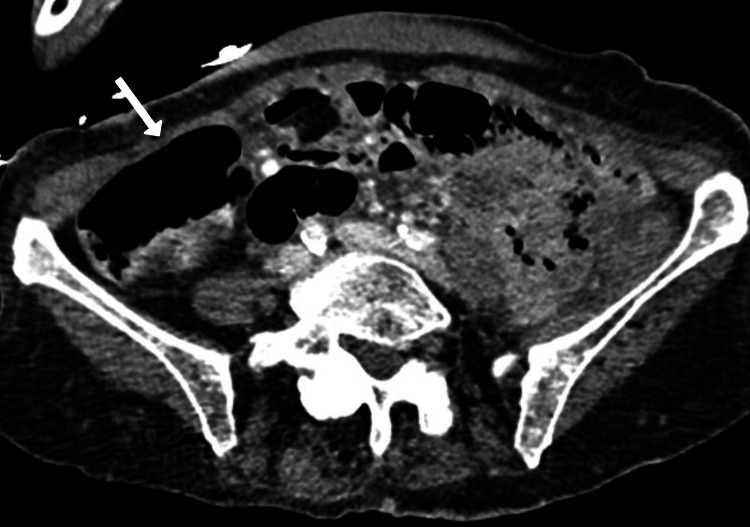
Axial contrast-enhanced computed tomography (CT) of the abdomen and pelvis demonstrating severe colitis characterized by extensive wall thickening and edema involving the distal transverse, descending, and sigmoid colon. The colon proximal to these segments appears unremarkable. No small bowel dilation, diverticulosis, or abnormal mesenteric lymphadenopathy is seen.

On initial examination, the patient was tachycardic (114 beats per minute), tachypneic (25 breaths per minute), hypertensive (181/78 mmHg), afebrile with an oral temperature of 36.6 °C, and oxygenating well with a pulse oximetry reading of 99%. She appeared pale, distressed due to pain, had dry mucous membranes, and localized abdominal tenderness with guarding in the left lower quadrant. A rectal examination was negative for frank blood, and stool occult testing was also negative. On repeat examination one hour after presentation, the patient’s mental status was found to be altered (alert and oriented to zero (AOx0)) and she required 5 mg of haloperidol to manage her agitation. Laboratory results (Table 1) revealed leukocytosis, hypokalemia, elevated blood urea nitrogen, elevated total bilirubin, elevated troponin, and a urine drug screen positive for amphetamines, fentanyl, and tetrahydrocannabinol. Hemoglobin, hematocrit, creatinine, and liver function tests were all within reference range. Acetaminophen, salicylate, and ethanol levels were within normal limits. Lower extremity ultrasound showed no evidence of deep vein thrombosis. Computed tomography angiography (CTA) of the chest was negative for pulmonary embolism. Computed tomography (CT) of the head revealed encephalomalacia and mild chronic small vessel ischemic changes in the white matter. CT of the abdomen and pelvis with contrast demonstrated severe colitis, segmental bowel wall thickening, and edema involving the distal transverse, descending, and sigmoid colon (Figure 1). The patient met sepsis criteria and was given a 1.8-liter fluid bolus along with vancomycin, cefepime, and metronidazole.

**Table 1:** Laboratory Investigations on Admission

Test	Result	Reference Range
White blood cell count	22.1 × 10³/μL	4.0–11.0 × 10³/μL
Potassium	2.8 mmol/L	3.5–5.1 mmol/L
Blood urea nitrogen	29 mg/dL	7–20 mg/dL
Total bilirubin	1.2 mg/dL	0.1–1.0 mg/dL
Troponin	0.04 ng/mL	<0.01 ng/mL
Hemoglobin	13.9 g/dL	12.0–16.0 g/dL
Hematocrit	42.9 %	36–46 %
Creatinine	1.04 mg/dL	0.6–1.3 mg/dL
Aspartate aminotransferase	35 U/L	10–40 U/L
Alanine aminotransferase	30 U/L	7–56 U/L
Alkaline phosphatase	85 U/L	44–147 U/L
Albumin	4.1 g/dL	3.5–5.0 g/dL
Lactic acid	2.0 mmol/L	0.5–2.2 mmol/L

Gastroenterology was consulted, and ischemic colitis secondary to amphetamine use was considered the most likely diagnosis based on acute-onset left lower quadrant pain, diarrhea, melena, leukocytosis, and a positive urine drug screen, along with abdominal CT findings involving the distal transverse, descending, and sigmoid colon, which are classic watershed regions vulnerable to vasospasm. Recommendations included supportive care and encouragement of oral intake as tolerated. The patient was monitored as an inpatient over the next three days, during which she showed significant improvement in abdominal pain and a decrease in leukocytosis. She was discharged home in stable condition and followed up with her primary care provider one week later, at which time she continued to do well without complications.

## Discussion

Ischemic colitis is the most common form of intestinal ischemia, typically affecting elderly patients with vascular risk factors [1]. However, drug-induced ischemic colitis, particularly from amphetamines and methamphetamines, is increasingly recognized as a clinically significant but underappreciated etiology, especially in populations under 50 years old [2,5]. Recent pharmacovigilance data indicate that hospitalization rates for drug-induced ischemic colitis are approximately 47%, and mortality rates are about 10%, highlighting the seriousness of this condition [6]. These rates are comparable to those seen in ischemic colitis of all causes, where in-hospital mortality is reported at 11.5% and surgery rates at 17% [6]. Drug-induced cases are more prevalent in women and older adults, but severe outcomes, including death, are also reported in younger patients, particularly with amphetamine and methamphetamine use, where fulminant colonic necrosis and rapid clinical deterioration can occur [7].

Amphetamines and their analogs exert potent sympathomimetic effects, leading to intense vasoconstriction and microvascular compromise via increased catecholamine release and α1-adrenergic receptor-mediated vasospasm [3]. This pathophysiology parallels that of cocaine-induced ischemic colitis and can result in segmental or diffuse colonic ischemia, often involving watershed areas such as the splenic flexure and sigmoid colon [2,8]. While most reported cases occur in younger individuals, this case highlights that elderly patients with comorbidities and polysubstance use are also at risk, and the clinical presentation may be severe, with rapid progression to sepsis or multi-organ dysfunction [5,9,10]. Notably, the patient in this case presented at an age approximately two decades older than typically observed [2].

Medical management typically includes supportive care, antibiotics, intravenous fluids, bowel rest through fasting, blood pressure control, and addressing any precipitating factors, including discontinuing the drugs that caused the ischemic colitis [11]. Surgical resection of the infarcted bowel is necessary in approximately 20% of patients [11]. Persistent diarrhea or rectal bleeding for one to two weeks, postprandial pain, or failure to thrive following conservative treatment may also necessitate surgical resection [8]. In the absence of treatment, gastrointestinal perforation can occur and may be accompanied by sepsis [2,9]. Abscess formation or fulminant ischemic pancolitis can develop secondary to perforation, necessitating percutaneous drainage and colectomy, respectively [8].

Diagnosis of amphetamine-induced ischemic colitis relies on a high index of suspicion, particularly in patients with a history of stimulant use and without classic vascular risk factors [5]. Imaging typically reveals colonic wall thickening and mucosal edema, while endoscopic findings can range from mild mucosal changes to full-thickness necrosis [5,12]. Management is primarily supportive, with surgical intervention reserved for cases with transmural necrosis or perforation [8,11]. Most cases resolve with conservative therapy, but severe presentations can be fatal, as documented in the literature [5,9,12].

## Conclusions

This case underscores the importance of obtaining a thorough social and drug history in patients presenting with acute colitis, regardless of age, and maintaining clinical vigilance for atypical etiologies. It demonstrates that amphetamine-induced ischemic colitis, though usually reported in younger populations, can also occur in elderly patients with comorbidities and polysubstance use. Awareness of this potential cause can broaden differential diagnoses and support prompt, appropriate management to optimize recovery.
